# Non-invasive High Frequency Repetitive Transcranial Magnetic Stimulation (hfrTMS) Robustly Activates Molecular Pathways Implicated in Neuronal Growth and Synaptic Plasticity in Select Populations of Neurons

**DOI:** 10.3389/fnins.2020.00558

**Published:** 2020-06-16

**Authors:** Minoru Fujiki, Kelly Matsudaira Yee, Oswald Steward

**Affiliations:** ^1^Department of Neurosurgery, School of Medicine, Oita University, Oita, Japan; ^2^Reeve-Irvine Research Center, University of California, Irvine, Irvine, CA, United States; ^3^Department of Anatomy and Neurobiology, University of California, Irvine, Irvine, CA, United States; ^4^Department of Neurobiology and Behavior, University of California, Irvine, Irvine, CA, United States

**Keywords:** transcranial magnetic stimulation, high frequency burst, ribosomal protein S6, PI3K/Akt pathway, immediate early genes, c-fos, Arc, phospho-specific antibodies

## Abstract

Patterns of neuronal activity that induce synaptic plasticity and memory storage activate kinase cascades in neurons that are thought to be part of the mechanism for synaptic modification. One such cascade involves induction of phosphorylation of ribosomal protein S6 in neurons due to synaptic activation of AKT/mTOR and via a different pathway, activation of MAP kinase/ERK1/2. Here, we show that phosphorylation of ribosomal protein S6 can also be strongly activated by high frequency repetitive transcranial magnetic stimulation (hfrTMS). HfrTMS was delivered to lightly anesthetized rats using a stimulation protocol that is a standard for inducing LTP in the perforant path *in vivo* (trains of 8 pulses at 400 Hz repeated at intervals of 1/10 s). Stimulation produced stimulus-locked motor responses but did not elicit behavioral seizures either during or after stimulation. After as little as 10 min of hfrTMS, immunostaining using phospho-specific antibodies for the phosphorylated form of ribosomal protein S6 (rpS6) revealed robust induction of rpS6 phosphorylation in large numbers of neurons in the cortex, especially the piriform cortex, and also in thalamic relay nuclei. Quantification revealed that the extent of the increased immunostaining depended on the number of trains and stimulus intensity. Of note, immunostaining for the immediate early genes Arc and c-fos revealed strong induction of IEG expression in many of the same populations of neurons throughout the cortex, but not the thalamus. These results indicate that hfrTMS can robustly activate molecular pathways critical for plasticity, which may contribute to the beneficial effects of TMS on recovery following brain and spinal cord injury and symptom amelioration in human psychiatric disorders. These molecular processes may be a useful surrogate marker to allow optimization of TMS parameters for maximal therapeutic benefit.

## Introduction

Transcranial magnetic stimulation (TMS) is increasingly being used as a therapeutic intervention to enhance recovery following brain and spinal cord injury (Müller-Dahlhaus and Vlachos, 2013; Rodger and Sherrard, 2015; Zheng et al., 2020). TMS is also being tested as a potential replacement for electroconvulsive therapy for individuals with psychiatric disorders. Despite increasing use, the mechanisms through which TMS exerts therapeutic benefit is not established. One possibility is that benefits are due to enhancing neuronal activity during the stimulation, but there is evidence that can induce changes that endure long after the stimulation. For example, it has been reported that trains of pulses of TMS (repetitive TMS; rTMS) alter cortical excitability for hours after the stimulation period (Ziemann et al., 2008). In a critical review, Hoogendam et al. (2010) present seven lines of evidence suggesting that aftereffects of rTMS are due to induction of synaptic changes resembling long-term potentiation and depression (LTP and LTD). Evidence includes similarities in temporal patterns of stimulation required for induction, duration of changes, and sensitivity to pharmacological intervention. This and other evidence is consistent with the hypothesis that TMS can activate cellular and molecular mechanisms underlying different forms of synaptic plasticity such as LTP and LTD. Some of these forms of synaptic plasticity are considered as NMDA-R mediated (for a review, see Müller-Dahlhaus and Vlachos, 2013). Also, previous studies have documented that different patterns of TMS can activate immediate early gene (IEG) expression (c-fos and zif268) in select populations of neurons (Aydin-Abidin et al., 2008; Gersner et al., 2011; Volz et al., 2013), cause both increases and decreases in levels of different neuronal proteins involved in neurotransmitter function (Müller-Dahlhaus and Vlachos, 2013; Moretti et al., 2020), and activate expression of GFAP in astrocytes (Fujiki and Steward, 1997). Nevertheless, our understanding of consequences of different patterns of TMS is incomplete, both in terms of the molecular and cellular mechanisms that are activated and especially in terms of the populations of neurons in which TMS-induced molecular events occur.

To further explore the idea that the therapeutic benefits of TMS are due to enhancement of synaptic plasticity, one approach is to assess whether TMS can activate molecular pathways that have been implicated in activity-dependent plasticity. In this regard, signaling pathways involving kinase cascades are of particular interest because they can be assessed using immunocytochemical markers that identify neurons in which molecular cascades are activated. For example, recent studies have revealed that induction of LTP and learning experiences robustly activate phosphorylation of ribosomal protein S6 in neurons (Kelleher et al., 2004; Panja et al., 2009; Nihonmatsu et al., 2015; Pirbhoy et al., 2016; Puighermanal et al., 2017). Although there is evidence that phosphorylation of S6 may be part of the core mechanism underlying synaptic modifications during LTP (Kelleher et al., 2004), more broadly, S6 phosphorylation is a sensitive surrogate marker of activation of NMDA-receptor dependent signaling pathways that operate together to render synaptic and cellular changes underlying plasticity.

Immunostaining with phospho-specific antibodies provides a convenient tool to identify the populations of neurons in which S6 phosphorylation is activated. S6 phosphorylation is activated within minutes after a triggering event and can persist for hours (Pirbhoy et al., 2016). Because S6 phosphorylation is activated throughout the cell body and part of the dendritic tree, immunostaining provides information about the neuron types involved. In general, S6 phosphorylation can be activated by the AKT/mTOR pathway (Pende et al., 2004; Chowdhury and Köhler, 2015), but S6 phosphorylation in neurons can also be activated via MAP kinase/ERK 1-2 through NMDA receptor-dependent mechanisms (Roux et al., 2007; Pirbhoy et al., 2017). Of note, blockade of NMDA receptors with MK801 completely blocks synaptically-driven S6 phosphorylation in the dentate gyrus despite the fact that pharmacological evidence indicates that activation of phosphorylation is via both mTOR- and MAP kinase pathways (Pirbhoy et al., 2017).

Different antibodies are available that recognize different phosphorylation sites on S6 (Ser235-236 vs. Ser240-244). This provides an important tool to begin to identify upstream kinase pathways that are driving S6 phosphorylation. For example, phosphorylation at Ser240-244 is thought to be via mTOR-dependent signaling whereas phosphorylation of Ser235-236 can be via multiple signaling pathways including MAPK/ERK, PI3 kinase and mTOR (Gobert et al., 2008).

Based on the rationale supported by these previous data, the present study defines the pattern of activation of S6 phosphorylation as a consequence of high frequency TMS in rats. To link to previous mechanistic studies, we use the same pattern of stimulation that we have used in previous studies of synaptically-driven S6 phosphorylation in the dentate gyrus. Using phospho-specific antibodies for different phosphorylation sites (Ser235/236 and Ser240/244) for immunocytochemistry, we show that high frequency repetitive TMS (hfrTMS) at 400 Hz strongly activates S6 phosphorylation at both Ser235/236 and Ser240/244 in select populations of neurons in the cortex, especially the piriform cortex, as well as in thalamic relay nuclei. The extent of activation by hfrTMS is comparable to what can be achieved by direct electrical stimulation of the cortex at the same frequencies. Of note, comparisons of patterns of immunostaining for the immediate early genes Arc and c-fos reveal that both S6 phosphorylation and IEG expression are activated in many of the same populations of cortical neurons whereas thalamic neurons exhibit robust S6 phosphorylation with minimal if any induction of IEG expression. Taken together, our results expand understanding of the neuronal populations that are affected by TMS and document that TMS does activate kinase cascades that are strongly implicated in activity-dependent synaptic plasticity.

## Materials and Methods

### Animals and Experimental Protocol

Experiments involving hfrTMS were carried out at Oita University and were approved by the Oita University Ethical Review Committee. Experiments involving electrical stimulation of the cortex were done at the University of California Irvine (UCI) and were approved by the Institutional Animal Care and Use Committee (IACUC) at UCI.

Rats that received hfrTMS were adult male Sprague Dawley rats (body weight 290–375 g) that were housed at controlled room temperature (24.5–25.0°C) with a 12/12 h light/dark cycle and *ad libitum* food and water. The entire study was composed of 2 separate experiments involving 64 animals (20; time course for 60-bursts, 18; time course for 180-bursts animals, 16 for intensity study animals, 10 for sham controls; see Table 1 for details).

**TABLE 1:** Summary of animals.

Number of rats	Duration of stimulation	% Machine power intensity	Survival time	Motor response during hfrTMS
*N* = 3	10 min	50%	15 min	+++
*N* = 5	10 min	50%	30 min	+++
*N* = 3	10 min	50%	60 min	+++
*N* = 3	10 min	50%	120 min	+++
*N* = 3	10 min	50%	180 min	+++
*N* = 3	10 min	50%	360 min	+++
*N* = 3	15 min	50%	15 min	+++
*N* = 3	30 min	50%	30 min	+++
*N* = 3	30 min	50%	60 min	+++
*N* = 3	30 min	50%	120 min	+++
*N* = 3	30 min	50%	180 min	+++
*N* = 3	30 min	50%	360 min	+++
*N* = 3	0 min	0%	0–15 min	−
*N* = 3	10 min	18.75%	30–40 min	±
*N* = 3	10 min	25%	30 min	+
*N* = 3	10 min	75%	30 min	+++
*N* = 4	10 min	100%	30 min	+++
*N* = 10	10 min	45–50%/sham	15, 30–360, min	−

*Total 64 animals (20; time course for 60-bursts, 18; time course for 180-bursts animals, 16 for intensity study animals, 10 for sham controls). %Machine power intensity = % of maximal machine power intensity. Motor responses, + = forearm small contraction; ++ = medium contraction of extremities; +++ = strong contraction of whole body. ++; exhibited at 30–40% of machine power intensity, not in the list.*

### 400 Hz High Frequency Magnetic Stimulation

To deliver hfrTMS, rats were lightly anesthetized with urethane (1.5g/kg, i.p.) 10 min before starting the hfrTMS as previously described in Aydin-Abidin et al. (2008). Although transcranial magnetic stimulation is not painful, stimulating an awake rat while holding in position under the wand for 10 or 30 min is somewhat stressful, especially because of the loud sound generated by the magnetic stimulation device. Urethane was used for the hfrTMS-treated animals to avoid depressing effects of deep anesthesia as with Pentobarbital or ketamine (pilot studies revealed that rpS6 activation was completely abrogated by ketamine anesthesia; data not shown). Somesthetic stimulation by the hfrTMS was avoided by local anesthesia of skin and muscles of the face and neck regions with local injections of xylocain (4%, AstraZeneca, Wedel, Germany) 5 min prior to stimulation. To avoid direct visual interferences, the rat’s eyes were covered with dark plastic foil. Body temperature was monitored and maintained between 37 and 37.5°C.

**FIGURE 1:**
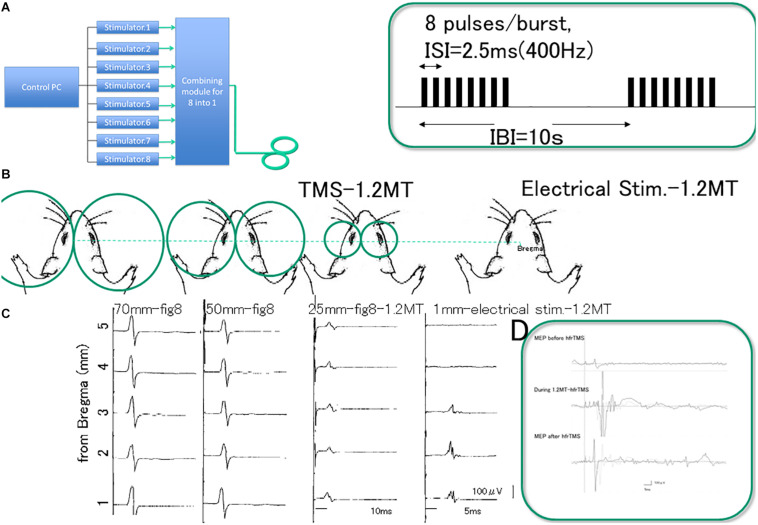
Experimental setting. Device allowing high frequency repetitive TMS (hfrTMS). **(A)** Illustrates a block diagram of the device that combines outputs from eight different stimulators to allow a burst of eight monophasic magnetic pulses at 400 Hz through a single coil (8 pulses delivered at 400 Hz, 20 ms duration at 10 s intervals). Burst patterns and stimulus pulse configurations were illustrated in the green box. G; Dimension difference between figure-8 coil diameters; we compared three different size (25, 50, and 70 mm) and direct motor cortical electrical stimulation (1 mm inter electrode distance) at 1.2 motor threshold (MT) of the motor evoked potentials (MEPs) under stereotactic frame **(B)**. Pilot study revealed that MEPs after single TMS with three different coil size were equivalent except for the small amplitude with 25 mm-figure-8 coil (**C**, third column) and qualitatively different from those after direct motor cortical electrical stimulation (C fourth column). MEP recordings indicate sharp-compound muscle responses during hfrTMS **(D)**. Note that 8 pulses/burst delivered at 400 Hz, 20 ms duration burst evokes amplitude facilitation during hfrTMS. Note; Green box indicate stimulus pulse interval (2.5 ms of inter stimulus interval: ISI, 10 s of inter burst interval: IBI) and EMG during stimulation. Eight pulses at 400 Hz activate the motor cortex based on observable motor responses involving the limbs (noted in Methods) and MEP. Each single burst consisted of 8 pulses at 1.2MT (50%) intensity resulted in long duration-single-compound muscle responses during hfrTMS. TMS, transcranial magnetic stimulation; MT, motor threshold; ISI, inter stimulus interval; IBI, inter burst interval; MEP, motor evoked potential; hfrTMS, high frequency repetitive transcranial stimulation.

Preliminary studies were undertaken to test three different size coils (25, 50, and 70 mm) delivering magnetic pulses at 1.2 the motor threshold (MT) of the motor evoked potentials (MEPs). MT was determined by decreasing stimulator output by 1% machine output until MEPs disappeared and then increasing the output in 1% increments until six MEPs of 50 μV peak-to-peak were elicited out of every 12 trains of single monophasic wave pulses. For this study, rats (*n* = 8) were anesthetized and placed in a stereotactic frame (Figure 1B). Recording methods were similar to what has previously been described (Brus-Ramer et al., 2007; Fujiki et al., 2010; Hsieh et al., 2012; Sykes et al., 2016; Tang et al., 2016). For comparison, other rats (*n* = 3) were prepared similarly and received direct electrical stimulation of the motor cortex. For this, a craniectomy was done over the motor cortex and stimulating electrodes spaced 1 mm apart were positioned at different locations in the motor cortex. Electric stimulation yielded mMEPs from the forelimb muscle when the motor cortex was stimulated 2 mm anterior, 2–3 mm lateral to bregma.

As reported Sykes et al. (2016), a 25 mm-figure-8 coil, placed over the rat’s scalp can be systematically adjusted to the best position for eliciting MEP via the motor cortex. The threshold for activation of the muscles was somewhat higher and the MEP amplitudes were smaller with the 25 mm-figure 8 coil than with the other coils but the outcomes were largely similar. On the other hand, basic waveforms of the MEPs from the forelimb muscles were similar regardless of the coil diameter, and the optimal position for magnetic stimulation was over the motor areas with the midpoint of the figure-8 coil at 2 mm lateral to bregma (center of gravity).

Although an ideal-size animal coil design for equivalent spatial resolution has been proposed (Tang et al., 2016), the fact that the smallest coil available is still too large for rat’s head is a current limitation. Thus, we chose the 70 mm-figure-8 coil because it minimized coil over-heating problems with HFS (Figure 1C).

For the main study, 400 Hz-hfrTMS was delivered via the 70 mm figure-8 coil positioned over the rat’s head with the rat in a stereotactic frame. The midpoint of the coil was centered 2 mm lateral from bregma above the left motor cortex unilaterally. It should be noted that different patterns of rTMS induce different aftereffects (Di Lazzaro and Rothwell, 2014) and that monophasic and biphasic TMS activate preferentially different cortical circuits (Di Lazzaro et al., 2018). Pulses were monophasic based on previous studies in humans of optimal pulse waveform (Nakamura et al., 2016). High frequency magnetic bursts were generated via a set of 8 separate magnetic stimulators (Magstim200^2^; The Magstim Co. Ltd) connected with a specially designed combining module (Figure 1). This device combines outputs from eight stimulators to allow a train of eight monophasic magnetic pulses at 400 Hz through a single coil. Except where noted (Table 1) stimulus amplitude was set at an intensity that evoked muscle twitch of the extremities (MT were distributed between 33 and 41%; typically 1.2 MT requires ranging 39.6–49.2%, 46.7 ± 2.7% of maximal strength, some groups were stimulated at below MT (18–25%) and above 1.2MT (50–100%) for intensity study; see Supplementary Table S1). Motor responses during hfrTMS, approximately 1000 μV were equivalent between with 1.2MT (50%) and above 1.2MT (75–100%) (*t* = 0.161, *P* > 0.05; Figure 1D and Supplementary Material). The stimulator delivers monophasic current pulses 200 μs in duration. The switching elements transfer up to 225 joules per pulse to the coil depending on the intensity setting. At the intensity setting of 70%, peak magnetic flux at the center of the coil is approximately 1.63 Tesla. The peak induced voltage gradient is approximately 6 V/cm, and the calculated charge density/phase is 1–2 μ coulombs/cm^2^ (Figure 1).

The stimulation paradigm was based on the patterns used to induce perforant path LTP, specifically delivery of trains of pulses (8 pulses at 400 Hz, 20 ms duration) at 10 s intervals for 60 or 180 hfrTMS trains. We chose this stimulation paradigm so as to link to our previous studies documenting activation of S6 phosphorylation in the dentate gyrus, which dissected signaling pathways and explored molecular mechanisms (Pirbhoy et al., 2016, 2017). It should be noted that different patterns of stimulation are used to induce LTP in hippocampal slices including theta burst stimulation and stimulation with 100 Hz trains. However, consequences of these patterns of stimulation on S6 phosphorylation have not been extensively explored, and so we went with the 400 Hz paradigm where data on neurotransmitters and signaling mechanisms have been partially elucidated (Pirbhoy et al., 2017). The most extensive stimulation (180 trains) involved delivery of hfrTMS trains over a 30-min period, which is sufficient to robustly induce S6 phosphorylation in the dentate gyrus (Pirbhoy et al., 2016). Our previous studies involving perforant path LTP (Pirbhoy et al., 2016) document that urethane anesthesia does not interfere with the induction of S6 phosphorylation.

To evaluate the time course of increases in rpS6 expression, rats that had received 60 (10 min) stimulus trains were killed humanely by anesthetic overdose 15, 30, 60, 120, 180, and 360 min after the initiation of the stimulation (3 rats per condition). Rats that received 180 (30 min) stimulus trains were killed 30, 60, 120, 180, and 360 min after the initiation of the stimulation. Measures were compared with controls that received no stimulation (time = 0, *n* = 3). Rats were perfused with 4% paraformaldehyde, brains were sectioned in the coronal plane, and sections were stored in buffer prior to immunostaining.

Rats in the sham stimulation-treated control group (*n* = 3) were anesthetized and positioned on the same stereotactic frame as hfrTMS animals but the stimulation coil was positioned 8 cm above the rat’s head so that no stimulation was delivered, but the click sound associated with each pulse was still present. Rats were euthanized 30 min post-sham stimulation.

To evaluate the effect of stimulus intensity on the increases in rpS6 expression, animals that had received 60 (10 min) of stimulus trains at different stimulator output 0, 18.75, 25, 50, 75, and 100% of machine output were killed humanely by anesthetic overdose 30 min post-stimulation (3 animals per condition), and prepared for immunohistochemistry for rpS6.

### Electrical Stimulation of the Cortex-400 Hz Trains, 8 Pulses/Train

*In vivo* physiological experiments involved adult Fischer rats of both sexes. Rats were anesthetized with urethane and placed in a stereotaxic apparatus. The scalp was incised and machine screws were placed in burr holes at 2.0A, 2.0L and 4.0P, 3.0L from bregma. Leads from a stimulus isolation unit were attached to the screws, stimulus intensity was set so that single pulses elicited forepaw twitch on the side contralateral to the stimulation, and then 400 Hz trains (8 pulses per train) were delivered every 10 s for 10 min (480 pulses in 60 trains). Rats were allowed to survive for 20, 30, or 40 min after the initiation of stimulation, and then received Fatal Plus and were perfused with 4% paraformaldehyde. Brains were prepared for IHC as below. Data from these animals have also been included in another study (Steward et al., 2020).

### Immunocytochemistry

Immunohistochemical study and analysis was performed at Reeve-Irvine Research Center, University of California, Irvine. Brains were sectioned in the coronal plane at 40 μm thickness on a Vibratome^®^. Free-floating sections were placed in microfuge tubes in nanopure water and tubes were then placed in a boiling water bath for 5 min for antigen retrieval. Sections were then immunostained using the following antibodies (Table 2). Phospho-S6 ribosomal protein (Ser235/236), Rabbit mAb (1/250 dilution, Cell Signaling Technology, catalog number #4858; Phospho-S6 ribosomal protein (Ser240/244, 1-250, Cell Signaling Technology catalog number #2215), polyclonal rabbit anti c-fos (1:1000, Millipore; ABE457), and polyclonal rabbit anti-Arc (1:1000; Synaptic Systems; 156-003).

**TABLE 2:** List of Antibodies.

Antibody	Antigen	Species	RRID	Dilution	Manufacturer
Phospho- ribosomal protein S6 (Ser235/236)	Synthetic phosphopeptide corresponding to residues surrounding Ser235/236 of human ribosomal protein S6	Rabbit monoclonal	4858	1:250	Cell Signaling Technology
Phospho- ribosomal protein S6 (Ser240/244)	Synthetic phosphopeptide corresponding to residues surrounding Ser240/244 of human ribosomal protein S6	Rabbit polyclonal	2215	1:250	Cell Signaling Technology
c-fos	Peptide mapping to the N-terminus of human c-fos	Rabbit polyclonal	ABE457	1:1000	Millipore
Arc	Strep-Tag^®^ fusion protein of full-length mouse arc	Rabbit polyclonal	156-003	1:1000	Synaptic Systems
Secondary Biotin-SP AffiniPure F(ab’)_2_ Fragment Donkey Anti-Rabbit IgG (H+L)	Rabbit whole molecule IgG (H+L)	Donkey polyclonal	711-066-152	1:500	Jackson ImmunoResearch Laboratories, Inc.
catalyzed reporter deposition (CARD) amplification using tyramide-Cy3 for immunofluorescence					

Sections were incubated for 16-20 hr in the primary antibody and then washed and incubated in the secondary antibody (Biotin-SP conjugated donkey anti-rabbit IgG; 1:500; Jackson ImmunoResearch; 711-066-152) for 1–2 h followed by washes and incubation in ABC-HRP (Vector Laboratories; PK-6100). For the mouse monoclonal antibody, the secondary antibody was a horse anti-mouse IgG used at a dilution of 1:100 in 5% normal horse serum. For the rabbit polyclonal antibody against c-fos, the secondary antibody was donkey ant-rabbit IgG, was used at a dilution of 1:100 in normal goat serum. Sections were then washed with TBS, mounted on 0.5% gelatin subbed slides and cover slipped with Vectashield^®^.

For immunofluorescence, free-floating sections underwent the same antigen retrieval procedure described above except that following incubation in ABC-HRP, sections were stained by catalyzed reporter deposition (CARD) amplification using tyramide-Cy3 as the substrate. Primary antibodies were phospho-S6 ribosomal protein-ser235/236 (1:250; Cell Signaling Technology; #4858), Arc antibody (1:1000; Synaptic Systems; #156-003), Secondary antibodies were as above. Following the amplification of Cy3 by CARD, sections were washed in TBS, mounted on 0.5% gelatin subbed slides and cover slipped with Vectashield^®^.

For quantitative assessment of immunostaining, optical density (OD) measurements were taken across the cortex using an M4 Microcomputer Imaging Device (MCID), Imaging Research. Digital images were collected at 400×. The light intensity was adjusted so that areas exhibiting background levels of labeling (the white matter of the corpus callosum) were just above threshold, whereas areas exhibiting maximal levels of labeling (the pyramidal cell layer, i.e., layer V of the motor cortex) were within the measuring range. Then, a series of OD measurements were taken across all layers of the cortex from the cortical surface to the white matter with a 20 μm × 20 μm measuring frame (see Figure 7). A row of five separate measurements were taken at each level and the OD values at each level (called row numbers in the figures) were averaged. The values in the graphs illustrate the mean and standard deviation of the five measurements.

### Statistical Analysis

All data are represented as mean ± standard error of the mean (SEM). Different groups of animals were compared using one-way (two-way for time course) ANOVA with the Student-Newman-Keul *post hoc* analysis (SPSS, Cary, NC, United States). Experiments with three or more groups were analyzed with a two-way ANOVA, followed by the *post hoc* Bonferroni-Dunn test. Differences were considered significant at *P* ≤ 0.05.

## Results

### Immunostaining for pS6 in Anesthetized Un-Stimulated Rats

**FIGURE 2:**
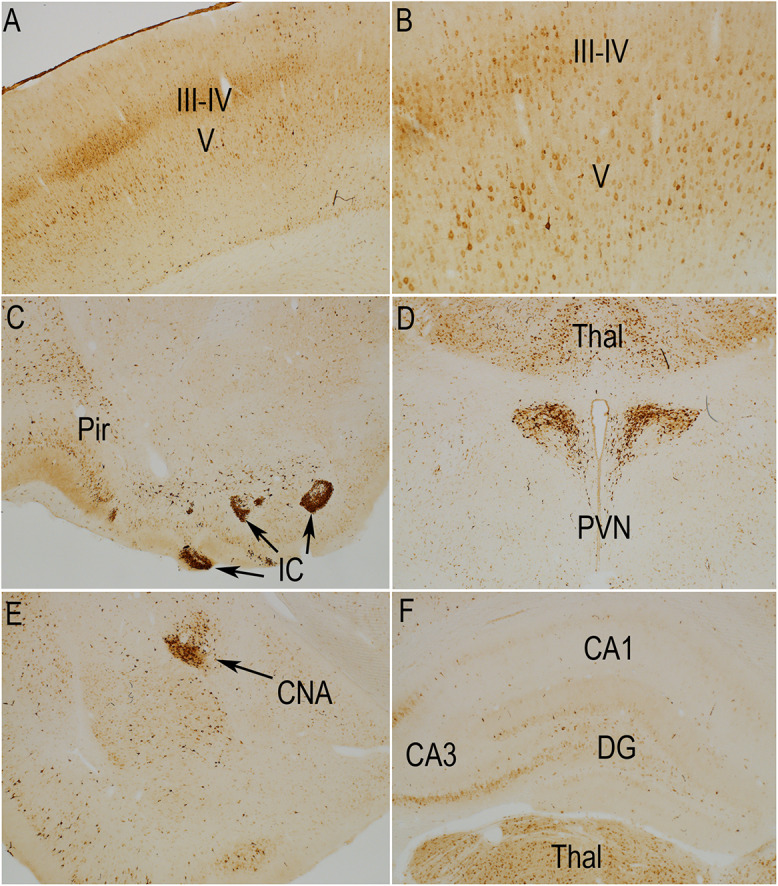
Distribution of phosphorylation of ribosomal protein S6 (pSer-235/236) in a sham stimulation-treated control animal. The panels illustrate increases in immunostaining for phospho-S6 in rats that had been anesthetized and were exposed to 400 Hz click sound with the stimulation coil positioned 8cm above the rat’s head (sham stimulation). **(A,B)** p-S6 staining in layer V; **(B)** Higher magnification of the same section shown in **(A)**. Piriform cortices (Pir; **C**), thalamus (Thal) and paraventricular nucleus of the hypothalamus (PVN; **D**), central nucleus of the amygdala (CNA; **E**) and the hippocampus and granule cell layer **(F)**. Levels of immunostaining for rpS6 at p-Ser235/236 were low except for Islands of Calleja (IC; **C**) and paraventricular nucleus of the hypothalamus (**D**; PVN). Pir, Piriform cortices; IC, Islands of Calleja; Thal, Thalamus; PVN, paraventricular nucleus; CNA, central nucleus of the amygdala; DG, dentate gyrus.

**FIGURE 3:**
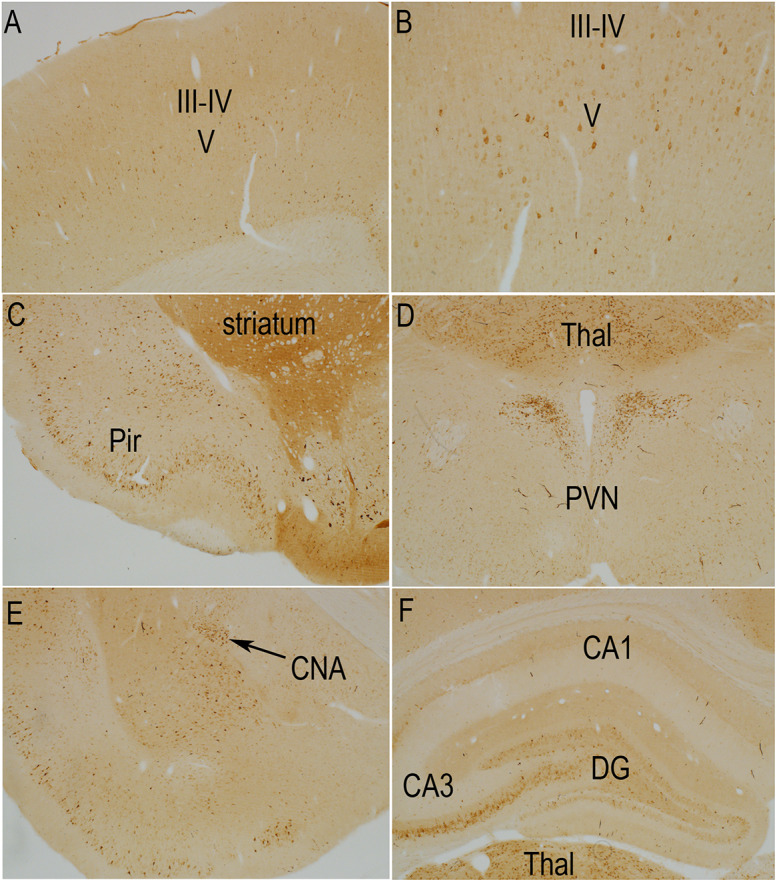
Distribution of phosphorylation of ribosomal protein S6 (pSer-240/244) in a sham stimulation-treated control animal. The panels illustrate increases in immunostaining for phospho-S6 in rats that had been anesthetized and were exposed to 400 Hz click sound with the stimulation coil positioned 8cm above the rat’s head (sham stimulation). **(A,B)** p-S6 staining in layer V; **(B)** Higher magnification of the same section shown in A. Piriform cortices (Pir; **C**), thalamus (Thal) and paraventricular nucleus of the hypothalamus (PVN; **D**), central nucleus of the amygdala (CNA; **E**) and the hippocampus and granule cell layer **(F)**. Levels of immunostaining for p-Ser-240/244 was higher overall in the striatum **(C)** and over neuropil layers such as some of the laminae of the hippocampus and dentate gyrus **(F)**. Pir, Piriform cortices; Thal, Thalamus; PVN, paraventricular nucleus; CNA, central nucleus of the amygdala; DG, dentate gyrus.

Previous studies have documented that S6 phosphorylation is activity- and experience-dependent. For example, S6 phosphorylation is robustly induced with strong synaptic activation as with induction of perforant path LTP and by behavioral experience (Pirbhoy et al., 2016, 2017). Prolonged anesthesia leads to some reduction in S6 phosphorylation over time compared to what is seen if animals are euthanized and perfused immediately (unpublished observations). Accordingly, to interpret changes induced by TMS, Figures 2, 3 compare the pattern of immunostaining for the two phospho-specific antibodies in rats that had been anesthetized and were exposed to 400 Hz click sound with the stimulation coil positioned 8cm above the rat’s head (sham stimulation).

In rats perfused after anesthesia and sham stimulation, overall levels of immunostaining for rpS6 at p-Ser235/236 were low. There were relatively few p-Ser235/236 positive neurons in most brain regions including the cerebral cortex (Figures 2A,B). The overall pattern of light labeling of a small number of neurons is consistent with our previous observations in rats that had been anesthetized for 30 min or more (not shown).

Nevertheless, certain populations of neurons did exhibit moderate to high levels of immunostaining under anesthesia (Figure 2). For example, there was robust immunostaining of neurons in the Islands of Calleja (Figure 2C, IC) and paraventricular nucleus of the hypothalamus (Figure 2D, PVN). There was moderate staining of neurons in the thalamic ventro medial – lateral posterior nuclei (Figures 2C,F, Thal), pyriform cortex (Figure 2C, Pir) and central nucleus of the amygdala (CNA, Figure 2E, CEM). In the sensory areas of the neocortex, small neurons in layer III-IV were lightly stained (Figure 2A). The overall number, location in layers III–IV, and differential distribution in sensory vs. non-sensory cortical areas suggests these are likely to be cortical neurons that receive input from the thalamus. There were scattered pS6-positive neurons in other cortical layers, including layer VI. Of note, a narrow column of neurons in the auditory cortex exhibited moderate levels of staining for pS6, likely due to the 400 Hz clicks delivered by the hfrTMS apparatus (not shown).

As described elsewhere (Pirbhoy et al., 2016), some neurons in the CA3 region of the hippocampus exhibited moderate levels of immunostaining and there were a few labeled neurons in the pyramidal cell layer of the hippocampus, especially CA3, and granule cell layer of the dentate gyrus (Figure 2F). As noted in previous studies, the number of S6-positive neurons in the hippocampus and other brain regions, and intensity of labeling of individual neurons increases in response to behavior (Pirbhoy et al., 2016; Steward et al., 2020).

The pattern of immunostaining was somewhat different for p-Ser-240/244 although many of the same populations of neurons were labeled (Figure 3). For example, immunostaining for p-Ser-240/244 was higher overall in the striatum (Figure 3C), and neurons in the central nucleus of the amygdala, that were labeled for p-Ser-235/236 were not strongly labeled for p-Ser-240/244 (Figure 3E). There was also a distinct laminar pattern of immunostaining over the neuropil layers of the hippocampus and dentate gyrus (Figure 3F). For example, immunostaining in stratum radiatum of the hippocampus and the inner 1/3 of the molecular layer of the dentate gyrus was conspicuously lighter than stratum lacunosum-moleculare of the hippocampus, the outer 2/3 of the molecular layer of the dentate gyrus, and the hilus and stratum lucidum. Of note, this pattern corresponds to the pattern of termination of different pathways. Stratum lacunosum-moleculare of the CA1 region of the hippocampus and the outer 2/3 of the molecular layer of the dentate gyrus are innervated by the perforant path. The hilus of the dentate gyrus and stratum lucidum is the site of termination of mossy fibers from dentate granule cells. This selective laminar pattern invites the speculation that basal levels of Ser-240/244 phosphorylation are determined the different afferent populations.

We note here only some aspects of differential labeling under resting conditions.

The selective staining of certain populations of neurons is of course of interest, but full description would require a manuscript of its own. In addition, as we and others have reported elsewhere, there is activation of phosphorylation of different populations of neurons as a consequence of behavioral experience, including simply removing the animal from its home cage. Thus, full descriptions of differential staining would have to include animals that were killed under different conditions.

### Electrical Stimulation of the Cortex Robustly Activates rpS6 Phosphorylation Over Widespread Regions

To provide data against which to compare results of TMS, we first assessed consequences of unilateral electrical stimulation of the cortex using patterns of stimulation that are typically used to LTP in perforant path projections to the dentate gyrus (10 pulse trains at 400 Hz at 1/10 s intervals). We chose this paradigm to link to our previous studies documenting that this pattern of stimulation induces robust phosphorylation of ribosomal protein S6 in target neurons in the dentate gyrus (Pirbhoy et al., 2016, 2017). Rats received a total of 60 400 Hz trains over the course of 10 min via skull screws and were perfused 20, 40, and 60 min post- stimulation.

**FIGURE 4:**
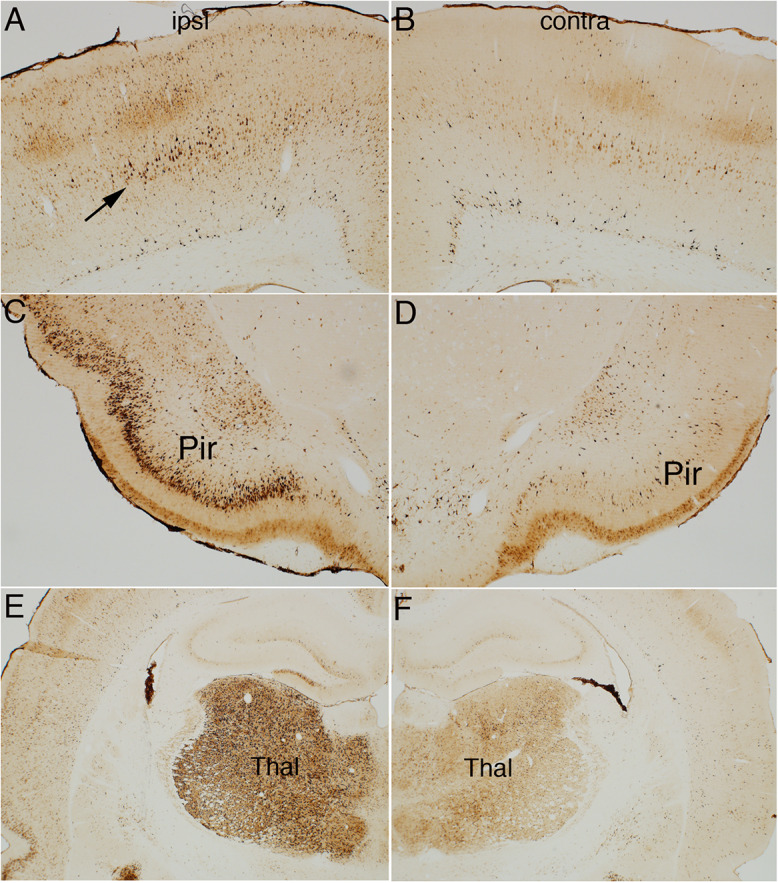
Direct cortical electrical stimulation activates phosphorylation of ribosomal protein S6 (pSer-235/236). The panels illustrate increases in immunostaining for phospho-S6 after high frequency direct cortical electrical stimulation of one side of the cortex in a rat. **(A)** p-S6 staining in layer V ipsilateral to the stimulation; **(B)** contralateral side of the same section shown in **(A)**. **(C,D)** Piriform cortices (Pir) ipsilateral **(C)** and contralateral **(D)** to direct motor cortical electrical stimulation. **(E,F)** thalamus (Thal) ipsilateral **(E)** and contralateral **(F)** side of the same section shown in **(E)**. Pir, Piriform cortices; Thal, Thalamus; ipsi, ipsilateral; contra, contralateral.

**FIGURE 5:**
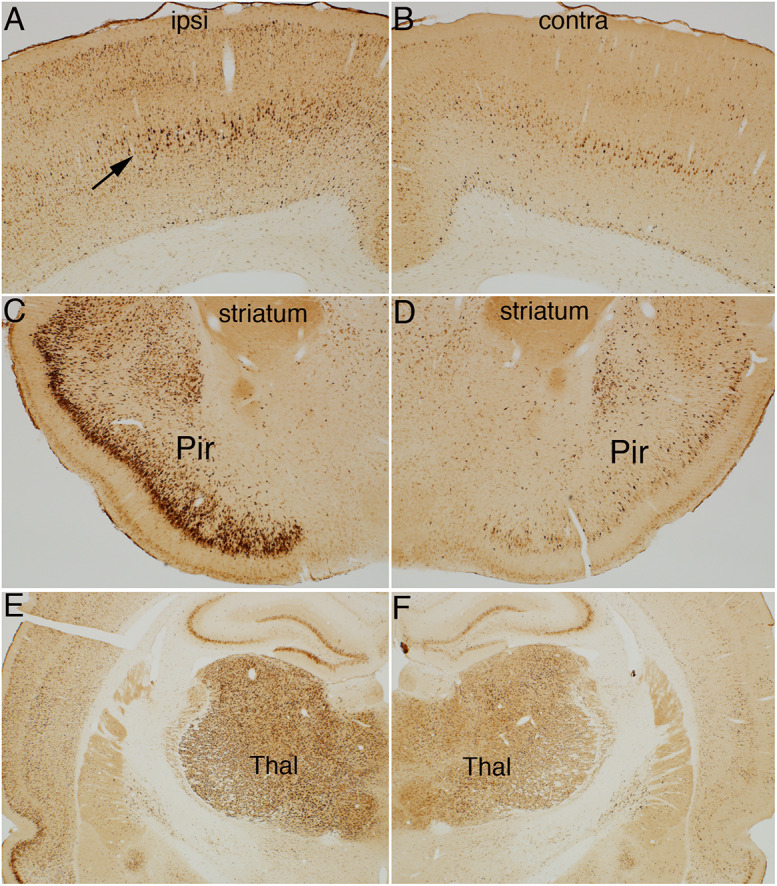
Direct cortical electrical stimulation activates phosphorylation of ribosomal protein S6 (pSer-240/244). The panels illustrate increases in immunostaining for phospho-S6 after high frequency direct cortical electrical stimulation of one side of the cortex in a rat. **(A)** p-S6 staining in layer V ipsilateral to the stimulation; **(B)** contralateral side of the same section shown in **(A)**. **(C,D)** Piriform cortices (Pir) ipsilateral **(C)** and contralateral **(D)** to direct motor cortical electrical stimulation. **(E,F)** thalamus (Thal) ipsilateral **(E)** and contralateral **(F)** side of the same section shown in **(E)**. Pir, Piriform cortices; Thal, Thalamus; ipsi, ipsilateral; contra, contralateral.

Figure 4 illustrates the pattern of immunostaining for pSer-235/236 in a rat that was perfused 40 min after the start of the 10 min long stimulation period. In the sensorimotor cortex, pSer-235/236 phosphorylation was induced on the side of the stimulation in many neuron types across cortical layers especially large pyramidal neurons in layer V, which are the cells of origin of the corticospinal tract (Figure 4A, arrow) in comparison to the contralateral side (Figure 4B). Increased immunostaining was evident throughout the rostro-caudal axis extending from the rostral-most tip of the frontal lobe through the entorhinal cortex posteriorly. pSer-235/236 phosphorylation was robustly induced in neurons in layer II and III on the side of the stimulation (Figure 4C) in comparison to the contralateral side (Figure 4D). Of note, there was strong activation of pSer-235/236 phosphorylation in thalamic relay nuclei that project to the dorsal neocortex on the side of the stimulation (Figure 4E) in comparison to the contralateral side (Figure 4F). Similar patterns of pSer-235/236 immunostaining were seen at 20 and 60 min (not shown). Immunostaining for p-Ser 240/244 revealed strong activation by direct electrical stimulation in a pattern similar to what is seen with p-Ser 235/236 (Figure 5).

Activation of S6 phosphorylation was completely blocked in rats that were anesthetized with ketamine/xylazine that received 400 Hz electrical stimulation (data not shown). This strongly suggests that activation of phosphorylation is due to synaptic activity and NMDA-receptor activation consistent with previous findings for activation of S6 phosphorylation in dentate granule cells with HFS of the perforant path (Pirbhoy et al., 2016).

### Cortical Stimulation Also Induces IEG Expression Over Widespread Regions

**FIGURE 6:**
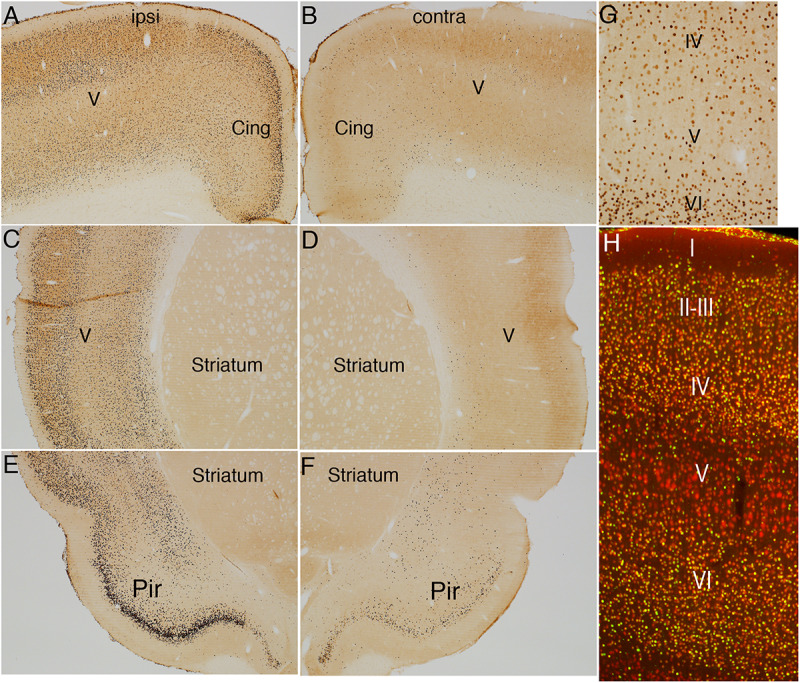
Direct cortical electrical stimulation activates immediate early gene c-fos. The panels illustrate increases in immunostaining for c-fos after high frequency direct cortical electrical stimulation of one side of the cortex in a rat. **(A)** c-fos staining in layer V ipsilateral to the stimulation; **(B)** contralateral side of the same section shown in **(A)**. **(C,D)** Striatum level ipsilateral **(C)** and contralateral **(D)** side of the same section shown in **(C)**. **(E,F)** piriform cortices (Pir) ipsilateral **(E)** and contralateral **(F)** to direct motor cortical electrical stimulation. **(G,H)** NeuN-positive neurons in most cortical layers were positive for c-fos whereas most large neurons in layer V did not express c-fos at detectable levels. Pir, Piriform cortices; Cing, cingulate cortices; ipsi, ipsilateral; contra, contralateral.

Our previous studies of activity-dependent S6 phosphorylation in the dentate gyrus have shown that intense synaptic activity induces immediate early gene (IEG) expression in many of the same neurons in which S6 phosphorylation is activated (Pirbhoy et al., 2016). To assess whether electrical stimulation of the cortex would induce IEG expression in the same populations of neurons in which S6 phosphorylation was activated, we immunostained sections for c-fos and Arc protein (Figure 6).

**FIGURE 7:**
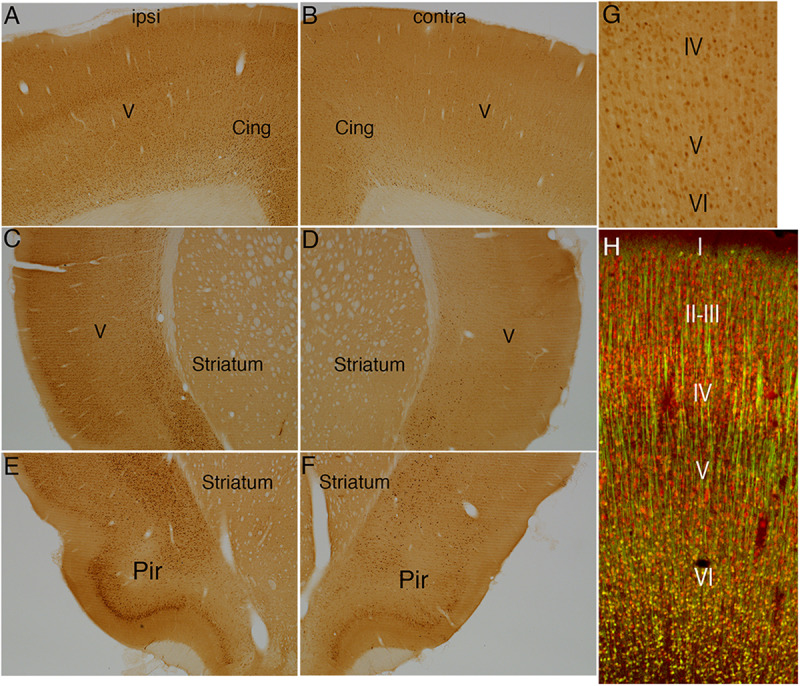
Direct cortical electrical stimulation activates immediate early gene Arc. The panels illustrate increases in immunostaining for Arc after high frequency direct cortical electrical stimulation of one side of the cortex in a rat. **(A)** Arc staining in layer V ipsilateral to the stimulation; **(B)** contralateral side of the same section shown in **(A)**. **(C,D)** Striatum level ipsilateral **(C)** and contralateral **(D)** side of the same section shown in **(C)**. **(E,F)** piriform cortices (Pir) ipsilateral **(E)** and contralateral **(F)** to direct motor cortical electrical stimulation. **(G,H)** Note that only the smaller neurons in layer V were Arc-positive whereas Arc was not induced in large pyramidal neurons in layer V. Pir, Piriform cortices; Cing, cingulate cortices; ipsi, ipsilateral; contra, contralateral.

Surprisingly, the pattern of induction of IEG expression was different than what was seen with pS6 activation, and also differed between the two IEGs. Unilateral cortical stimulation induced c-fos expression in virtually all areas of the cortex on the side of the stimulation including areas in the dorsal cortex such as the cingulate cortex and sensorimotor cortex (Figure 6A vs. Figure 6B) and ventral areas including the piriform cortex and throughout the rostro-caudal axis of the cortex (Figure 6C vs. Figure 6D; note that these are the same cases described in Steward et al., 2020).

Although c-fos was induced in large numbers of neurons in cortical layers II-IV and layer VI, there were very few c-fos positive neurons in layer V (Figure 6G). To document this directly, sections were co-immunostained for c-fos and NeuN to mark neurons (Figure 6H). NeuN-positive neurons in most cortical layers were positive for c-fos whereas most large neurons in layer V did not express c-fos at detectable levels. Of note, and in contrast to what was seen with pS6, there was no induction of c-fos in thalamic neurons; neurons in certain nuclear groups in the thalamus were c-fos positive, but the pattern of labeling was comparable on the two sides of the brain (data not shown).

The pattern of induction of Arc expression was similar to that of c-fos with a few exceptions. As with c-fos, there was robust induction of Arc protein throughout the cortex on the side of the stimulation with large numbers of neurons in cortical layers II-IV showing induced expression. In contrast to c-fos, there were also numerous Arc-positive neurons in layer V (Figure 7A). Higher magnification views revealed that only the smaller neurons in layer V were Arc-positive whereas Arc was not induced in large pyramidal neurons in layer V (Figures 7G,H). Together, these results reveal that direct electrical stimulation of the cortex activates S6 phosphorylation and IEG expression over a widespread area on the side of the stimulation, but that different neuron types exhibit different patterns of activation.

### HfrTMS Activates S6 Phosphorylation in a Pattern Similar to What Is Seen With Direct Electrical Stimulation of the Cortex

During high frequency magnetic stimulation, there were visible muscle contractions associated with each stimulus train (8 pulses/burst, 2.5 ms of inter stimulus interval; Figure 1), but there was no evidence of abnormal activity between or after stimulus trains (no abnormal EMG firing during, no EEG/EMG activity after stimulation period). Muscle responses were greater with increasing stimulus intensity (namely 50, 75, 100% Machine power intensity). MEP recordings indicate sharp-compound muscle responses during hfrTMS (see Figure 1D). Note that 8 pulses/burst delivered at 400 Hz, 20 ms duration burst evokes amplitude facilitation during hfrTMS.

**FIGURE 8:**
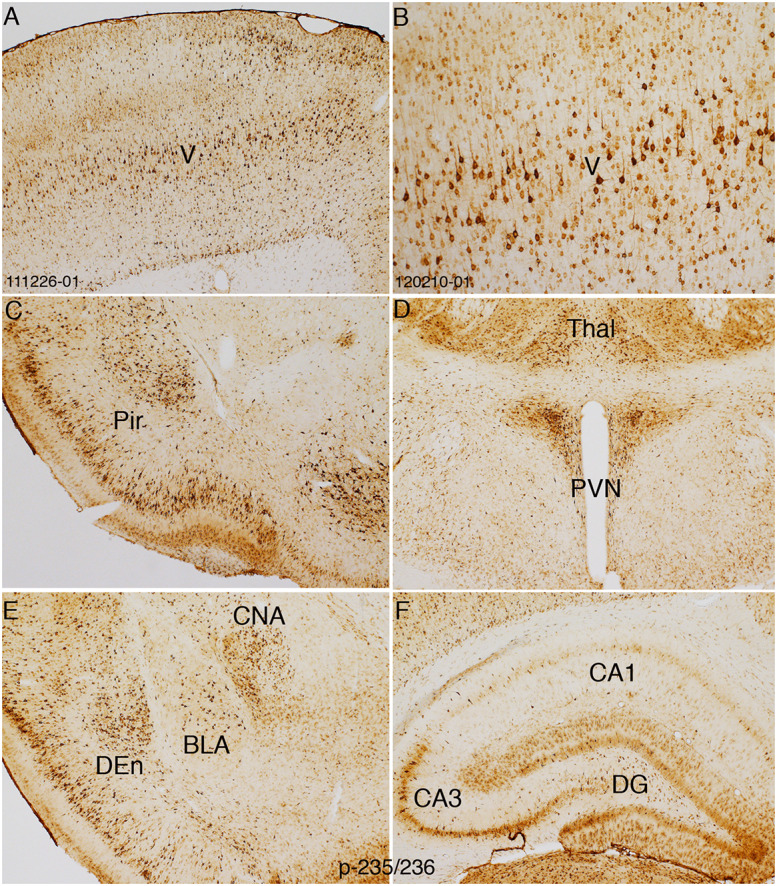
HfrTMS leads to comparable selective localization of rpS6 p-235/236 immunostaining with direct cortical electrical stimulation. Distribution of rpS6 p-235/236 immunostaining neuronal cells as reveled by immunocytochemistry in an animal following 60 hfrTMS (400 Hz, 10 s intervals) trains in the pyramidal cell layers in the sensorimotor cortices **(A,B)**, piriform cortices (Pir; **C**), paraventricular nucleus (PVN) and thalamus (Thal; **D**), basolateral nucleus of the amygdala (BLA), dorsal endopiriform nucleus (DEn), central nucleus of the amygdala (CAN; **E**) and the hippocampus and granule cell layer **(F)**. Pir, Piriform cortices; Thal, Thalamus; PVN, paraventricular nucleus; CNA, central nucleus of the amygdala; BLA, basolateral nucleus of the amygdala; DEn, dorsal endopiriform nucleus; DG, dentate gyrus.

In contrast to direct electrical stimulation of one side of the cortex, hfrTMS activates the brain bilaterally, so there is no intra-animal control. Accordingly, assessing changes in S6 phosphorylation requires comparisons across cases using sections prepared for IHC at the same time. Figure 8 illustrates patterns of immunostaining for p-Ser-235/236 in a rat that received 60 trains of 400 Hz magnetic stimulation at 50% machine power that was immunostained in the same run as the case illustrated in Figure 2. In the rat that received hfrTMS, there were increased numbers of p-Ser-235/236-positive neurons in different layers of the sensorimotor cortex including the large pyramidal neurons of layer V (Figures 8A,B) and dramatically higher levels of p-Ser-235/236 immunostaining of neurons in layers II and III of the piriform cortex in comparison to the control (Figure 8C). Increased numbers of p-Ser-235/236-positive neurons were seen bilaterally. Levels of immunostaining for p-Ser-235/236 in the PVN appeared comparable to control. At more caudal levels, there were increases in immunostaining for p-Ser-235/236 in the basolateral nucleus of the amygdala (BLA) and dorsal endopiriform nucleus (DEn) (Figure 8E) whereas immunostaining in the central nucleus of the amygdala appeared (CNA) comparable to control. There were increases in the immunostaining for p-Ser-235/236 in the granule cell layer (GCL) of the dentate gyrus and also in the molecular layer, which contains the dendrites of dentate granule cells and in the pyramidal cell layer of the hippocampus, especially in area CA3.

There was some case-to-case variability in the pattern of increased immunostaining. For example, only about 50% of the cases exhibited increases in immunostaining in the molecular layer of the dentate gyrus (data not shown). It is likely that the effective depth of stimulation with hfrTMS may vary across cases leading to variability in activation in deep brain structures like the hippocampus. There were no obvious areas of damage in any brain region, although sections were not stained for H&E.

### Activation of rpS6 Phosphorylation in Astrocytes

**FIGURE 9:**
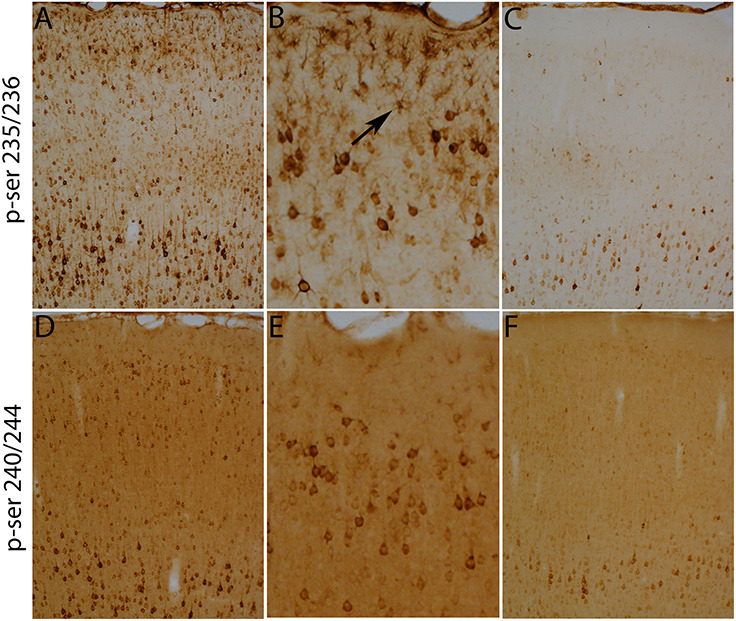
Qualitative analyses of the increases in immunostaining of rpS6 at ser-235/236 and ser-240/244. Immunostaining for rpS6 p-240/244 were qualitatively similar to what was seen with rpS6 p-235/236. The main exception was that astrocytes were not obviously labeled for rpS6 p-240/244 (compare **A,B** with **D,E**). **(B,E)** Illustrate the pattern of immunostaining at higher magnification in the cerebral cortex layer I-III in rpS6 p-235/236 **(B)** and rpS6 p-240/244 **(E)** in the same animals. Scale bar represents 400 μm in **(A,C,D,F)**, 100 μm in **(B,E)**.

Notably, there were also increases in immunostaining for p-Ser-S6-235/236 in small cells in layer I of the cortex with the morphological appearance of astrocytes (small cells with multiple processes). Figures 9A,B illustrates one case 30 min post-stimulation in which activation of s6 phosphorylation in cells in layer I was especially evident. Although these cells have the morphology of astrocytes, definitive identification requires co-immunostaining with an astrocyte-specific marker. Activation of S6 phosphorylation (p-ser 235/236) is most evident in astrocytes in layer I (Figure 9A), but there may be some p-S6 positive astrocytes in other layers that are less evident because of the prominence of p-S6 positive neurons.

At 6 h, immunostaining of astrocytes was no longer evident (Figure 9C). In this regard, we have previously shown that a different pattern of hfrTMS up-regulates expression of GFAP in astrocytes within the first few hours after stimulation (Fujiki and Steward, 1997).

### HfrTMS Activates Phosphorylation of rpS6 at Both Ser-235/236 and Ser-240/244

Generally, the results seen when immunostaining for p-Ser-240/244 were similar to what was seen with p-Ser-235/236. The main exception was that astrocytes were not obviously labeled for p-Ser-240/244 (Figures 9D–F).

### Time Course of Increase in rpS6 Expression Following TMS

**FIGURE 10:**
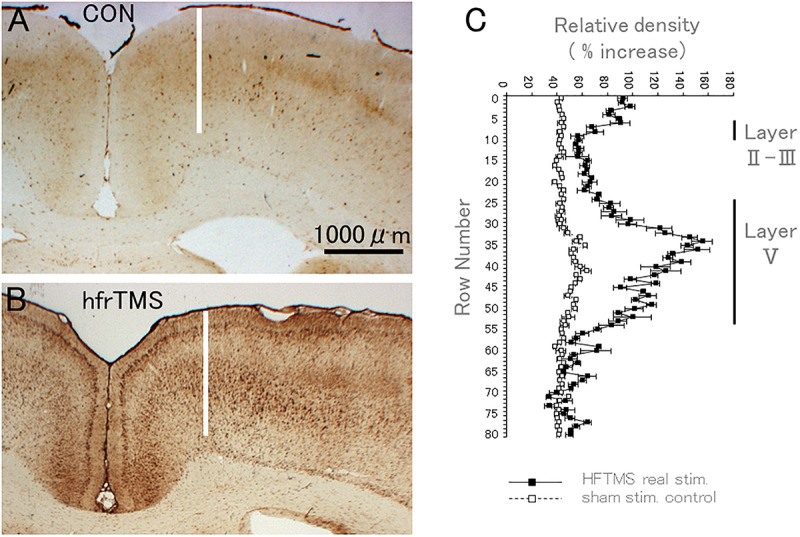
HfrTMS leads to selective localization of rpS6 immunostaining in activated sensorimotor cortex. **(A)** Distribution of rpS6 immunostaining neuronal cells as reveled by immunocytochemistry in a sham stimulation-treated control animal. **(B)** Distribution of rpS6 immunostaining neuronal cells as reveled by immunocytochemistry in an animal following 60 hfrTMS (400 Hz, 10 s intervals) trains. **(C)** Graph illustrating the average optical density (OD) of labeling across the pyramidal cell layer, i.e., layer V of the motor cortex in the case illustrated in control **(A)** and in real stimulated animal **(B)**. Bars indicate the standard deviation of the five measurements at each level. CON, control; hfrTMS, high frequency repetitive transcranial stimulation.

To quantify increases in immunostaining for rpS6 p-Ser-235/236 over time and in response to different stimulation paradigms, we first quantified levels of immunostaining across cortical layers by assessing optical density (OD). Figure 10 illustrates this analysis in the anesthetized control rat that received click stimulation only, vs. a rat that received repeated 60 trains (60 bursts; 10 min duration) of 400 Hz hfrTMS at 100% machine power (2X higher than for the case illustrated in Figure 4).

**FIGURE 11:**
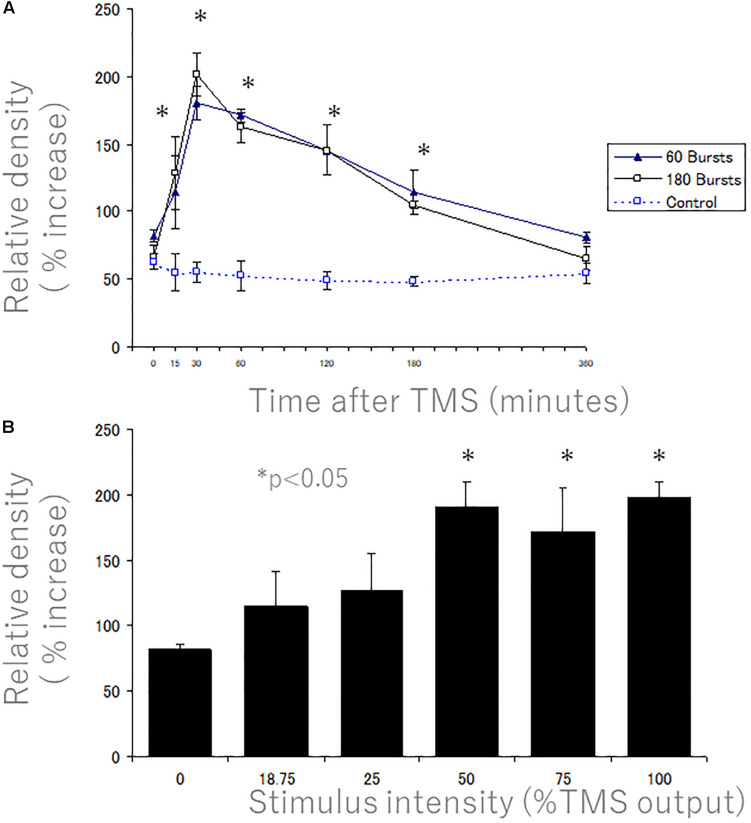
Quantitative time course and stimulus intensity analyses of the increases in rpS6 levels after 400 Hz-hfrTMS as revealed by immunocytochemistry. Quantitative analyses of the increases in rpS6 levels after 400 Hz high frequency magnetic stimulation as revealed by immunocytochemistry. **(A)** The graph illustrates the average optical density in the layer V of the motor cortex 0, 15, 30, 60, 120, 180, and 360 min after 60 bursts (solid triangles) and 180 bursts (blank squares) of high frequency magnetic stimulation (treatment effect: *P* = 0.0003). The values represent the mean and standard deviation of the measurements. There were no significant differences between 60 bursts (10 min duration) and 180 bursts (30 min duration). **(B)** The graph illustrates the average optical density in the layer V of the motor cortex 30 min after high frequency magnetic stimulation at 0, 18.75, 25, 50, 75, and 100% of machine output (con. vs. 50%; *P* < 0.05; con. vs. 75%; *P* < 0.05; control vs. 100%; *P* < 0.008). The values represent the mean and standard deviation of the measurements. There is a sharp change in the extent of rpS6 phosphorylation between 25 and 50% stimulus intensity. A * denotes *p* < 0.05 [repeated measures two way ANOVA].

To quantify increases at different time points, we plotted the peak optical density (Figure 11 above) at different times post-stimulation (Figure 11A). Peak levels of p-235/236 were at 30 min, and then gradually decreased, returning to near control levels by 6 h (Figure 11A). Two-way ANOVA revealed a significant difference over time (*P* = 0.0003). *Post hoc* comparisons by SPSS (Cary, NC, United States) revealed that levels were significantly higher than unstimulated control at all time points except 6 h (asterisks in Figure 11A indicate significance). There were no significant differences between 60 bursts (10 min duration) and 180 bursts (30 min duration) in the time course of induction of immunostaining for p-Ser-235/236 (Figure 11A).

### Effect of Stimulus Intensity on TMS-Induced rpS6 Expression

We assessed whether activation could be increased by increasing stimulus intensity. Figure 11B illustrates the relationship between the stimulus intensity and the extent of the upregulation of rpS6 levels in the motor cortex as determined by the peak in OD in the plots across cortical layers. Rats that received hfrTMS at 50% machine setting or higher showed dramatic activation of S6 phosphorylation (con. vs. 50%; *P* < 0.05; con. vs. 75%; *P* < 0.05; control vs. 100%; *P* < 0.008). Activation was less dramatic in rats that received stimulation at less than 50% output (approximately 1.2–1.3-fold; not significantly different than control). Thus, there appeared to be a sharp change in the extent of rpS6 phosphorylation between 25 and 50% stimulus intensity.

### Comparison of Immunostaining for rpS6-235/236 vs. the IEGs c-Fos and Arc

**FIGURE 12:**
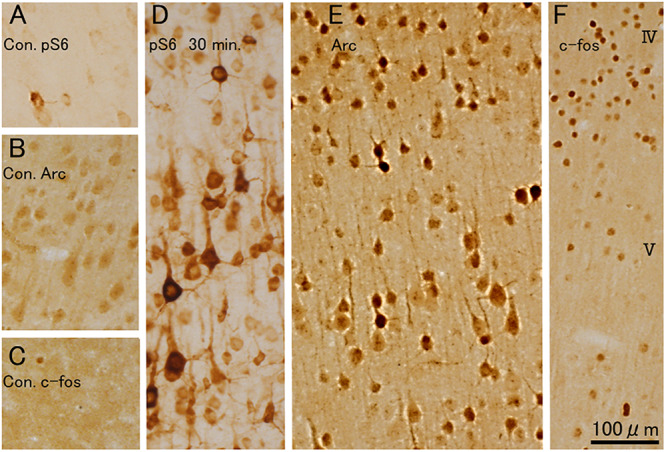
400 Hz-hfrTMS leads to increases the co-activated rpS6-positive and Arc, c-fos protein-positive neurons in the motor cortex. **(A–C)** Pattern of immunostaining for rpS6 in sham control animal **(A)**. Pattern of immunostaining for Arc **(B)** and c-fos **(C)** in sham control animal. **(D–F)** Pattern of immunostaining for rpS6 **(D)**, Arc **(E)** and c-fos **(F)** 30 min, after 400 Hz hfrTMS. Note that many neurons including some pyramidal neurons in layer V were strongly positive for rpS6 and Arc protein, the larger pyramidal neurons in layer V were less intensely stained for Arc and that the distribution of c-fos did not align with the distribution of Arc-positive pyramidal neurons within the layer V.

With direct cortical stimulation, the pattern of rpS6 in individual neurons is different from what is seen with IEG transcription (c-fos). To assess whether this differential pattern is also seen with hfrTMS, we compared the pattern of labeling for c-fos and Arc protein with what is seen for rpS6 (p-Ser-235/236) (Figure 12). We focused on the motor cortex especially in layers II and V, which contains the cell bodies of pyramidal neurons. In sham controls, there were only a few rpS6-positive or Arc, c-fos protein-positive neurons (Figures 12A–C, respectively). Thirty minutes post-stimulation, many neurons including some pyramidal neurons in layer V were strongly positive for rpS6 and Arc protein (Figures 12D,E). However, the larger pyramidal neurons in layer V were less intensely stained for Arc. Smaller neurons in more superficial layers were also positive for rpS6 and Arc. Many c-fos positive neurons were also evident (Figure 12F), but their distribution did not align with the distribution of Arc-positive pyramidal neurons within the layer V. In particular, c-fos positive neurons were abundant in layers II–IV and layer VI, but were rare in layer V (the same pattern seen with direct cortical stimulation).

## Discussion

Our results demonstrate that non-invasive hfrTMS strongly induces phosphorylation of rpS6 and activates IEG expression in widespread areas of the cortex. The fact that the pattern of activation is similar to what is seen with high frequency electrical stimulation of the dorsal surface of the cortex suggests that S6 phosphorylation is triggered by propagated synaptic activity rather than directly by the currents generated by the stimulation. In what follows, we discuss the approach and possible mechanisms and functional significance.

### Novel Technology Allowing Delivery of High Frequency Transcranial Magnetic Stimulation

Magnetic stimulation offers considerable advantages as a non-invasive and apparently innocuous way to manipulate neuronal activity *in vivo*. Previous studies document that magnetic stimulation can indirectly modulate neuronal gene expression (Fujiki and Steward, 1997; Aydin-Abidin et al., 2008), and here we show that it can also strongly activate rpS6 phosphorylation, which is a hallmark of activation of AKT-mTOR and IEG expression.

The device provides unique advantages for magnetically induced monophasic-400 Hz high frequency stimulation which has previously been impossible. Commercially-available stimulation devices cannot be driven at the high frequencies used here. The maximum stimulation frequency of most commercial devices is only 50 Hz with biphasic pulse polarity. This limitation has been overcome here by combining multiple individual monophasic units to drive a single wand. This technical innovation greatly broadens the scope of possibilities for non-invasive magnetic stimulation.

### HfrTMS Induces Rapid Phosphorylation of rpS6 in Neurons in Widespread Cortical Areas

Presumably hfrTMS preferentially activates the area of the cortex beneath the coil, depending on the eddy currents generated by the magnetic pulses. We show here, however, that hfrTMS induced robust phosphorylation of rpS6 in widespread areas of the cortex with seemingly comparable activation in dorsal regions (cingulate and sensorimotor cortex; layer V) and ventral regions (piriform cortex; layer II and III). A similar pattern was seen with direct electrical stimulation of the dorsal surface of the cortex. In the case of direct electrical stimulation, widespread activation throughout the cortex ipsilateral to the stimulation suggests that activation is not driven directly by currents generated at the site of stimulation. The most likely interpretation is that HFS triggers bursts of synaptic activation that propagate through the cortex. This interpretation is supported by previous studies that documented that HFS at 400 Hz that induces perforant path LTP induces rapid and prolonged phosphorylation of rpS6 in dentate granule cells that are completely blocked by local delivery of NMDA receptor antagonists (Pirbhoy et al., 2016). Also, our pilot studies indicate that activation of S6 phosphorylation by direct electrical stimulation of the cortex is completely blocked in rats anesthetized with ketamine-xylazine (ketamine is an NMDA receptor antagonist).

The important implication of our results is that even though TMS causes focal neuronal activation related to the exact pattern of current flow, there can be widespread molecular consequences, presumably driven by propagated synaptic activity. This provides a provocative explanation for the fact that functional consequences of hfrTMS that outlast the time of stimulation and may involve activation of plasticity-related signaling pathways at long distances from the actual areas of magnetic current flow.

Increases in immunostaining for pS6 indicate activation of phosphorylation of S6 protein, but there may also be increases in levels of ribosomal protein S6 (rpS6) itself due to the stimulation. For example, it has been reported that induction of LTP in hippocampal slices via delivery of brief 100 Hz trains is accompanied by protein synthesis-dependent increases in levels of rpS6 protein 30 min post-stimulation (Tsokas et al., 2007). In contrast, with 400 Hz stimulation of the perforant path, dramatic increases in immunostaining for pS6 were not accompanied by increases in levels of rpS6 protein (Pirbhoy et al., 2016). This question regarding detailed molecular mechanisms will require further study using models that are better suited for molecular analyses.

One caveat for our interpretations is that the actual patterns of magnetic current flow and resulting eddy currents are un-defined. Also, the patterns of neuronal activity that are induced are unknown, and may involve differential activation of neurons of different size or structure. Indeed, hfrTMS could activate inhibitory as well as excitatory circuits, so selective patterns of phosphorylation in different neuron groups could reflect the interplay between excitation and inhibition. Determining patterns of activation is technically challenging because with the current coils, the magnetic current flow occurs over a wide area, which interferes with physiological recording during the stimulation. In this regard, using activity-dependent markers such as S6 phosphorylation and IEG induction may actually provide a useful proxy measure.

### HfrTMS Induces Phosphorylation of rpS6 in Astrocytes

Following hfrTMS, small cells in layer I of the cortex with the morphology of astrocytes also stained for rpS6-235/236 but not rpS6240-244. This is noteworthy, because in our previous study involving HFS of the perforant path, there was no activation of rpS6 phosphorylation in astrocytes even when HFS was delivered for 2 h. In contrast, there was striking activation of S6 phosphorylation in astrocytes by a learning experience (Pirbhoy et al., 2016). Importantly, in Pirbhoy et al. (2016), the identity of pS6-positive cells was confirmed by co-immunostaining for GFAP. Although we did not carry out double-immunostaining for pS6 and GFAP in the present study, the morphology of the pS6-positive cells in layer I of the cortex was indistinguishable from that of the pS6-positive GFAP-positive astrocytes shown in Figure 8 in Pirbhoy et al. (2016).

The mechanisms underlying rpS6 phosphorylation in astrocytes remain to be defined. One possibility is direct activation of ionic currents in astrocytes at layer I because eddy currents triggered by TMS may be preferentially induced in superficial layers of the cortex. Another is that hfrTMS-triggered neuronal activity leads to the release of neurotransmitters that activate astrocytes via different intercellular signaling cascades than are activated by HFS of the perforant path. Phosphorylation at ser235/236 is activated by multiple signaling pathways including MAPK/ERK (Roux et al., 2007) and PKA (Gobert et al., 2008; Biever et al., 2015). In contrast, ser240/244 is predominantly mTOR-dependent, being phosphorylated by S6K1 and S6K2 (Pende et al., 2004). Thus, in the case of astrocytes, hfrTMS may activate one or more of the signaling pathways for which ser235/236 is a target but not mTOR.

### HfrTMS Induces IEG Expression in Many of the Same Neurons in Which S6 Phosphorylation Is Activated

HfrTMS also strongly induced expression of the immediate early genes c-fos and Arc in cortical neurons. Arc appeared to be induced in the same populations of neurons in which rpS6 phosphorylation was induced. However, c-fos was expressed in neurons with a somewhat different intracortical distribution. Further studies will be required to fully define differences in patterns of activation.

### The Possible Use of Non-invasive Magnetic Brain Stimulation as a Tool for Modulating Neuronal Regeneration

With hfrTMS, activation of S6 phosphorylation is especially prominent in large neurons in layer V in the sensorimotor cortex, which are the cells of origin of the corticospinal tract (CST). The degree of activation of rpS6 phosphorylation is comparable to that seen with conditional deletion of PTEN, which enables cortical motoneurons to regenerate their axons after SCI (Liu et al., 2010; Lewandowski and Steward, 2014; Danilov and Steward, 2015). With PTEN deletion, S6 phosphorylation is considered to be a downstream marker of persistent activation of mTOR (Liu et al., 2010). As discussed further below, this raises the interesting possibility that hfrTMS might be a useful way to use non-invasive techniques to drive molecular pathways that enhance regenerative growth after injury.

HfrTMS-60 bursts lead to peak induction of rpS6 phosphorylation at 30 min after stimulation, which then slowly returns to control levels. Essentially the same induction occurs with hfrTMS-180 bursts. These findings are compatible with the results of recent studies that showed a correlation between up-regulation of neurotrophic factors such as VEGF, known for being downstream of AKT-mTOR pathways and GFAP, induced by electrical stimulation in the rat cerebral cortices and hippocampus (Steward et al., 1997; Newton et al., 2003; Warner-Schmidt and Duman, 2007; Fujiki et al., 2010). The PI3K/Akt pathway is a central mediator in signal transduction pathways involved in cell growth, survival, and metabolism. Akt phosphorylates caspase 9 at Ser-196, thereby blocking cytochrome *c*-mediated caspase 9 activation *in vitro* (Cardone et al., 1998). Akt may rescue cells from apoptosis by inhibiting the Bax-dependent apoptosis pathway through a forkhead box transcription factor (Nakae et al., 2000).

Recent studies have shown that electrical stimulation of the motor cortex after spinal cord injury (SCI) enhances regenerative growth of corticospinal tract (CST) axons in the spinal cord and recovery of motor function (Brus-Ramer et al., 2007; Carmel et al., 2010, 2013, 2014; Carmel and Martin, 2014). The mechanism through which HFS promotes regenerative growth has not been defined. Our findings that both electrical stimulation and hfrTMS induce robust phosphorylation of S6 in the cells of origin of the CST (cortical motoneurons or CMNs) suggest the provocative idea that stimulation-induced CST growth might be a result of persistent activation of mTOR in CMNs.

Although direct electrical stimulation of the brain to promote motor recovery after SCI and other injuries to the motor system is potentially translatable, it would require neurosurgical implantation of stimulating electrodes. From the point of view of translatability, it would be better if it was possible to use non-invasive techniques such as hfrTMS.

One limitation, however, is that optimal parameters for TMS delivery are unknown. To assess TMS or any other intervention, it would be extremely valuable to have surrogate markers for the beneficial effects of functional stimulation. Surrogate markers are important because it would be extremely inefficient to test different stimulation paradigms by measuring regeneration or behavioral recovery because assessing either regeneration or recovery requires months to complete. To understand the underlying mechanisms and to verify compatibility with human and animal results, further experiments involving other patterns of stimulation such as theta burst stimulation will be informative. Safety may be a limitation because to our knowledge, 400 Hz-hfrTMS has never been employed in humans (Rossi et al., 2009). Comparisons between 400 Hz-hfrTMS and standard human protocols (such as 50 Hz-theta burst rTMS) would be most important in a future study. Hoogendam et al. summarize evidence that rTMS leads to LTD- or LTP-like aftereffects, depending on stimulus parameters, indicating bidirectional changes in synaptic efficacy (Hoogendam et al., 2010). On the other hand, Cirillo et al. (2017) review studies suggesting that rTMS leads to a broad range of long-lasting changes in neuronal function including LTP and LTD-like effects but other effects as well. Further detailed studies will be required to explore links between the neurobiological aftereffects induced by rTMS, hfrTMS and the induction of LTP/LTD and other forms of neuronal plasticity including changes in intrinsic properties of neurons.

Immunocytochemical studies of phosphorylation of S6 and activation of IEG expression may offer considerable advantages for future studies because the assays are straightforward and these are molecular pathways that are involved in synaptic plasticity and regenerative axonal growth. S6 phosphorylation may be especially relevant because it is a downstream marker for activation of multiple signaling pathways in neurons including mTOR, MAPK/ERK, and PI3 kinase (Gobert et al., 2008). The present results using antibodies for pS6 and IEGs for immunocytochemistry may represent a paradigm for rapid testing to compare TMS stimulation paradigms.

The degree of activation of rpS6 phosphorylation shown here is similar to what is seen with PTEN deletion, which enables neurons to regenerate their axons after spinal cord injury (Liu et al., 2010). In this situation, S6 phosphorylation is thought to reflect persistent activation of mTOR, which is known as a central regulator of cell growth during development (Ma and Blenis, 2009; Narayanan et al., 2009). Our findings raise the possibility that non-invasive hfrTMS might induce the same molecular changes in neurons as PTEN deletion. HfrTMS also induced expression of the immediate early genes Arc and c-fos and likely others, all of which may contribute to a growth-enabling phenotype. The present results define a paradigm to test whether repeated delivery of hfrTMS over periods of days would promote regenerative growth of CST axons and recovery of function after CST injury in a manner similar to direct electrical stimulation.

## Data Availability Statement

All datasets generated for this study are included in the article/Supplementary Material.

## Ethics Statement

The animal study was reviewed and approved by Ethical Committee of the School of Medicine, Oita University.

## Author Contributions

MF and OS designed the research and wrote the manuscript. MF, OS, and KY performed the research. MF analyzed the data.

## Conflict of Interest

OS was a co-founder and has economic interests in the company “Axonis,” which holds a license on patents relating to PTEN deletion and axon regeneration. The remaining authors declare that the research was conducted in the absence of any commercial or financial relationships that could be construed as a potential conflict of interest.
