# Supplementary material for: Non-invasive High Frequency Repetitive Transcranial Magnetic Stimulation (hfrTMS) Robustly Activates Molecular Pathways Implicated in Neuronal Growth and Synaptic Plasticity in Select Populations of Neurons
Source: Front Neurosci. 2020 Jun 16;14:558. doi: 10.3389/fnins.2020.00558 (PMC7308563; doi:10.3389/fnins.2020.00558)
Supplement: Supplementary file 1 [file Image_1.pdf]

<u>Suppl. Fig.1B.C.   Data for determine stimulus coil position using MEP</u>

[Materials and Methods; Third paragraph]

Preliminary studies were undertaken to test three different size coils (25, 50 and 70mm) delivering magnetic pulses at 1.2 the motor threshold (MT) of the motor evoked potentials (MEPs). MT was determined by decreasing stimulator output by 1% machine output until MEPs disappeared and then increasing the output in 1% increments until six MEPs of 50 µV peak-to-peak were elicited out of every 12 trains of single monophasic wave pulses. For this study, rats (n=8) were anesthetized and placed in a stereotactic frame (Fig. 1B). Recording methods were similar to what has previously been described (Brus-Ramer et al., 2007; Fujiki et al., 2010; Hsieh et al., 2012; Sykes et al, 2016, Tang et al., 2016). For comparison, other rats (n=3) were prepared similarly and received direct electrical stimulation of the motor cortex. For this, a craniectomy was done over the motor cortex and stimulating electrodes spaced 1mm apart were positioned at different locations in the motor cortex. Electric stimulation yielded mMEPs from the forelimb muscle when the motor cortex was stimulated 2mm anterior, 2-3 mm lateral to bregma.

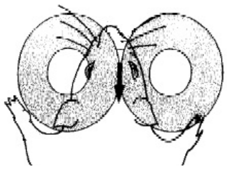

As reported Sykes et al., a <u>25mm-figure-8 coil, placed over the rat's scalp can be systematically adjusted to the best position for eliciting MEP via the motor cortex</u> (Sykes et al., 2016). The threshold for activation of the muscles was somewhat higher and the MEP amplitudes were smaller <u>with the 25 mm-figure 8 coil than with the other coils</u> but the outcomes were largely similar. On the other hand, basic waveforms of the MEPs from the forelimb muscles were similar regardless of the coil diameter, and the optimal position for magnetic stimulation was over the motor areas with the <u>midpoint of the figure 8 coil at 2mm lateral to bregma (center of gravity)</u>. Stereotactic TMS with experimental rat brain (OU-0363 and OU-0389_05882018JP) combines standard stereotactic frame and any size of TMS coil devices.

Although an ideal-size animal coil design for equivalent spatial resolution has been proposed (Tang et al., 2016), but the fact that the smallest coil available is still too large for rat's head is a current limitation. Thus, we chose the 70mm-figure-8 coil because it minimized coil over-heating problems with HFS (Fig. 1 C).

Suppl. Fig.1D   Data for MEP monitoring during hfrTMS

As an electrophysiological monitoring during hfrTMS motor evoked potential (MEP) were measured during, pre and immediately after hfrTMS only for intensity study animal, using a method described elsewhere (Fujiki et al., 2004). In brief, the MEP relies on the activation of subcortical structures by an electromagnetic coil placed over the cranium. TMS activates descending motor projections to motoneuron pools in the spinal cord. Our experiments assessed activation representative forelimb brachioradial muscles, MEPs were recorded via 27-gauge stainless steel needle electrodes inserted transcutaneously 5 mm apart. Signals were amplified and stored (Brain Amp DC, Brain Products, Germany, with 5-3000 Hz bandpass and 50 ms analysis time).

MEP amplitudes were measured in 16 animals for intensity study.
There were visible muscle contractions associated with each stimulus burst (consisted of 8 pulses/burst, 2.5ms of inter stimulus interval), getting greater whole body muscle responses according to increment of stimulus intensity (namely 50, 75, 100% Mache power intensity). Motor responses during hfrTMS, approximately 1000 μV were equivalent between with 1.2MT (50%) and above 1.2MT (75-100%) (t=0.161, P>0.05). Note that each single burst consisted of 8 pulses resulted in long duration-single-compound muscle responses during hfrTMS.
MEP during hfrTMS (stimulus artifact 2.5ms ISI, 8 pulses; 17.5 msec. duration of bursts/ train.

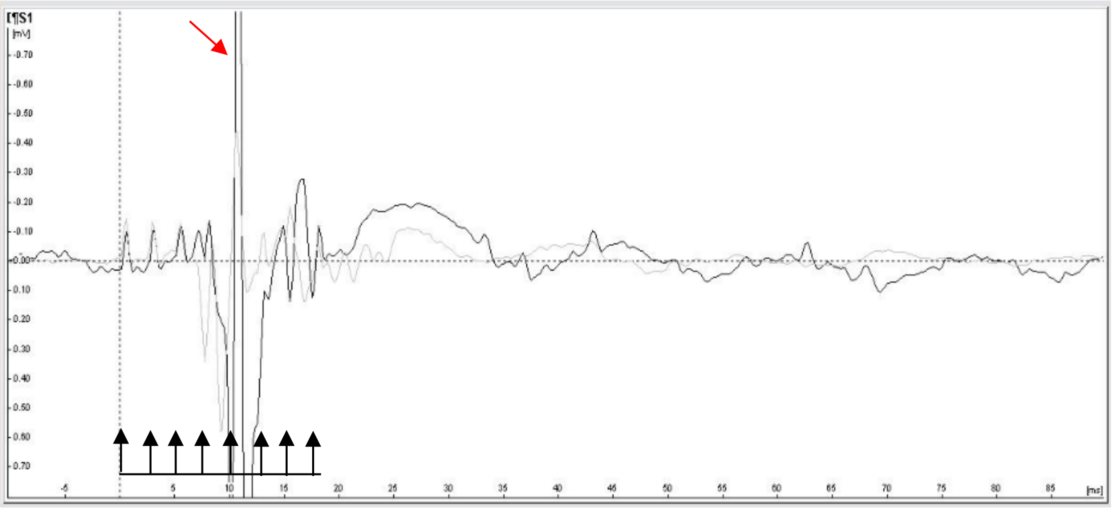

Supple. Figure 1D: MEP monitoring during hfrTMS (recorded from forelimb brachioradial muscle of animal 33).

As illustrated in this figure, muscle response elicited during single burst consisted of temporal summation of corticospinal muscle responses to each eight pulses.
