# Supplementary material for: Non-invasive High Frequency Repetitive Transcranial Magnetic Stimulation (hfrTMS) Robustly Activates Molecular Pathways Implicated in Neuronal Growth and Synaptic Plasticity in Select Populations of Neurons
Source: Front Neurosci. 2020 Jun 16;14:558. doi: 10.3389/fnins.2020.00558 (PMC7308563; doi:10.3389/fnins.2020.00558)
Supplement: Supplementary file 2 [file Table_1.docx]

**Suppl. Table 1 Correlation between stimulus intensity and Motor responses (MEP amplitudes)**

|  |  | MT(%) | 1.2MT(%) | Motor response | MEP(μV) during hfrTMS |
| --- | --- | --- | --- | --- | --- |
| 50%.10min_30min | 1 | 40 | 48 | +++ | 1028 |
|  | 2 | 33 | 39.6 | +++ | 1222 |
|  | 3 | 41 | 49.2 | +++ | 1251 |
| 50%.30min_30min | 4 | 38 | 45.6 | +++ | 1110 |
|  | 5 | 39 | 46.8 | +++ | 1031 |
|  | 6 | 40 | 48 | +++ | 1255 |
| 25%.10min_30min | 7 | 40 | 48 | + | 211 |
|  | 8 | 41 | 49.2 | + | 152 |
|  | 9 | 39 | 46.8 | + | 312 |
| 75%.10min_30min | 10 | 41 | 49.2 | +++ | 1008 |
|  | 11 | 40 | 48 | +++ | 1111 |
|  | 12 | 38 | 45.6 | +++ | 1251 |
| 100%.10min_30min | 13 | 35 | 42 | +++ | 1421 |
|  | 14 | 41 | 49.2 | +++ | 1212 |
|  | 15 | 39 | 46.8 | +++ | 1118 |
|  | 16 | 38 | 45.6 | +++ | 1007 |
|  | Mean | 38.9375 | 46.725 |  |  |
|  | SD | 2.235136 | 2.682163 |  |  |
